# Supplementary material for: Mortality Related to Chronic Obstructive Pulmonary Disease during the COVID-19 Pandemic: An Analysis of Multiple Causes of Death through Different Epidemic Waves in Veneto, Italy
Source: Int J Environ Res Public Health. 2022 Oct 7;19(19):12844. doi: 10.3390/ijerph191912844 (PMC9565127; doi:10.3390/ijerph191912844)
Supplement: Supplementary file 1 [file ijerph-19-12844-s001.zip › ijerph-1921436-supplementary.pdf]

Table S1. Monthly age-standardized monthly mortality rate associated to COPD (European standard population 2013) observed in 2020 and percentage change with respect to rates predicted by SARIMA models based on 2008-2019 data, overall and by gender. Veneto region, Italy.

	Age-standardized rate, all	exp. from SARIMA	delta % obs/exp
Jan	8,8	11,0	-20,5
Feb	7,3	9,7	-24,8
Mar	9,7	8,9	8,6
Apr	10,7	6,8	58,4
May	6,7	6,8	-1,2
Jun	5,6	5,6	-1,5
Jul	6,4	5,6	14,8
Aug	6,0	5,3	11,5
Sept	6,2	5,3	17,8
Oct	7,6	6,6	15,6
Nov	11,3	6,8	65,4
Dec	13,7	7,6	79,2
**2020**	**100,0**	**86,2**	**16,0**

	Age-standardized rate, males	SARIMA	delta % obs/exp
Jan	15,0	16,0	-6,1
Feb	11,1	15,0	-26,1
Mar	14,1	14,7	-4,4
Apr	17,0	10,8	57,4
May	11,2	11,6	-3,1
Jun	8,3	8,6	-3,5
Jul	10,6	9,9	7,2
Aug	10,0	8,1	23,7
Sept	10,8	7,4	47,0
Oct	12,6	9,7	29,4
Nov	17,8	11,9	50,0
Dec	21,3	13,2	61,8
**2020**	**159,8**	**136,8**	**16,8**

	Age-standardized rate, females	SARIMA	delta % obs/exp
Jan	5,1	7,1	-27,8
Feb	5,0	6,6	-24,4
Mar	7,0	6,0	15,7
Apr	7,0	4,7	49,0
May	4,3	4,7	-8,1
Jun	3,8	3,7	0,7
Jul	4,0	3,8	6,3
Aug	3,7	3,8	-3,0
Sept	3,7	3,4	8,0
Oct	5,0	4,1	21,2
Nov	7,1	4,9	46,5
Dec	8,9	4,7	90,1
**2020**	**64,8**	**57,7**	**12,3**
